# How to erase memory traces of pain and fear

**DOI:** 10.1016/j.tins.2013.03.004

**Published:** 2013-06

**Authors:** Jürgen Sandkühler, Jonathan Lee

**Affiliations:** 1 Department of Neurophysiology, Center for Brain Research, Medical University of Vienna, Spitalgasse 4, A-1090 Vienna, Austria; 2 University of Birmingham, School of Psychology, Edgbaston, Birmingham B15 2TT, UK

## Abstract

Pain and fear are both aversive experiences that strongly impact on behaviour and well being. They are considered protective when they lead to meaningful, adaptive behaviour such as the avoidance of situations that are potentially dangerous to the integrity of tissue (pain) or the individual (fear). Pain and fear may, however, become maladaptive if expressed under inappropriate conditions or at excessive intensities for extended durations. Currently emerging concepts of maladaptive pain and fear suggest that basic neuronal mechanisms of memory formation are relevant for the development of pathological forms of pain and fear. Thus, the processes of erasing memory traces of pain and fear may constitute promising targets for future therapies.

## Highlights

• Currently emerging concepts of maladaptive pain and fear suggest that they share basic neuronal circuits and cellular mechanisms of memory formation.
• Recent studies have revealed processes of erasing memory traces of pain and fear that may be promising targets for future therapies.

## Memory traces of pain and fear

Memory traces of pain and fear are encoded by distinct but partially overlapping sets of synapses. For example, painful stimuli are highly effective for inducing fear learning [1]. Indeed, electric foot shock is the most commonly used outcome for fear-memory studies and it remains untested whether the mechanisms and principles outlined below apply equally to fear memories that do not involve activation of nociceptive pathways. However, acute and chronic pain are often associated with fear or anxiety [2–5]. Brain areas associated with fear, such as the amygdala and the cingulate and medial prefrontal cortices [6–8], are also relevant for the emotional/aversive and cognitive aspects of pain [9–12]. Here, we focus on forms of chronic pain and fear that involve the staged formation of enduring synaptic plasticity (Box 1). We discuss recent findings suggesting that some memory traces of pain and fear can be erased, which may provide novel options for future treatments.

## Memory traces of pain

Numerous clinically relevant conditions may change the properties and functions of the nociceptive system in ways that lead to: (i) the amplification of pain and the lowering of pain thresholds (hyperalgesia); (ii) spontaneous pain; (iii) spreading pain; and/or (iv) pain elicited by touch fibres (mechanical allodynia) (see Box 2 for definitions). Common causes include acute painful events (e.g., surgery, trauma, inflammation), drugs (e.g., opioids, chemotherapeutics), and diseases such as neuropathies, type I and type II diabetes, fibromyalgia, and sickness syndrome. The duration of pathological pain may exceed the duration of its primary cause by days to years and may involve synaptic plasticity at various sites in the nociceptive network (Box 3).

At present, only a few studies have specifically addressed the question of whether memory traces of pain can be erased under clinical conditions, but some treatments do appear to have lasting effects. Some forms of counter stimulation – such as transcutaneous electrical nerve stimulation, (electro-)acupuncture, and some forms of physical therapy – are reported to have analgesic effects that outlast the period of treatment in selected patients [13,14], but counterexamples exist, as described in [15,16].

### Induction, consolidation, and maintenance phases of lasting pain

#### Induction phase

Strong and/or lasting noxious stimuli trigger various neuroplastic changes in the central nervous system (CNS) including activity-dependent long-term potentiation (LTP) at the first synaptic relays in nociceptive pathways [17]. Paradoxically, similar ‘memory traces of pain’ can evolve in the absence of any noxious stimuli. For example, hyperalgesia and synaptic facilitation may develop during continuous application of opioids or on their abrupt withdrawal (opioid-induced hyperalgesia) [18–20] without the need for any concomitant stimulation of nociceptive nerve fibres.

#### Consolidation phase

**Figure 1:**
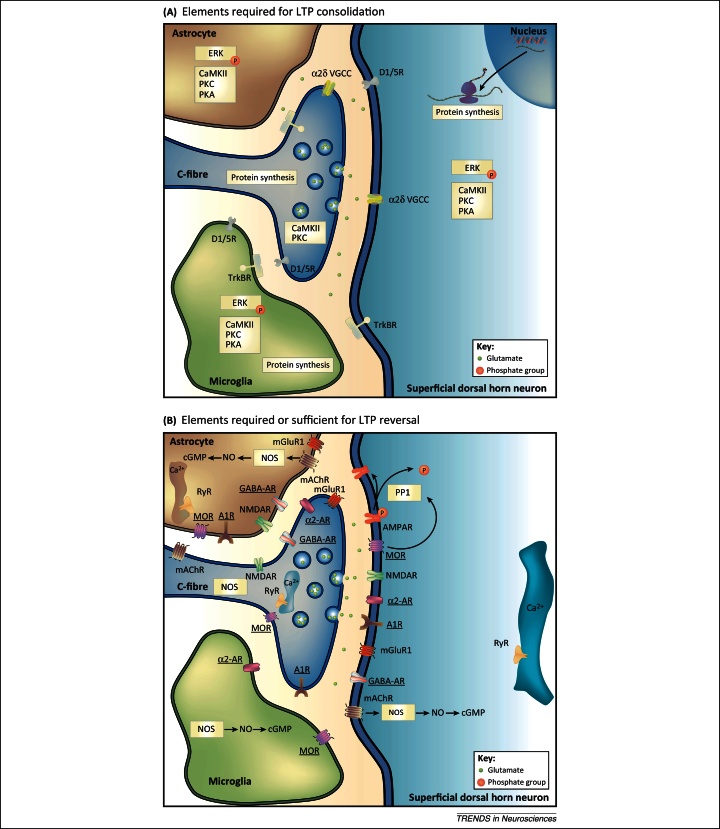
Signalling pathways of long-term potentiation (LTP) consolidation and LTP reversal at C-fibre synapses. The schemes summarise elements of signalling pathways that are required for the maintenance of LTP at spinal C-fibre synapses. Thus, when any of these elements is blocked, established LTP diminishes or disappears (required elements for LTP consolidation, **A**). (**B**) summarises elements that, when activated, reverse established LTP. These sufficient elements for the reversal of LTP are underlined. Elements that are not underlined are required for the reversal of LTP. When blocked, these elements prevent the reversal of LTP by at least one of the sufficient elements. Blockers and activators of the respective elements were usually applied topically to the spinal cord. Many of the known signalling elements are expressed at more than one cellular site, as shown in the figure. The cellular sites of action are thus unknown in most cases. Suggested signalling pathways are indicated by arrows. Modified from [122].

The development of chronic pain is an active process that requires time and which can be interrupted. The consolidation phase may last for hours to weeks. Some elements that are required for the consolidation of LTP in nociceptive pathways are shown in Figure 1A. Therapeutic interference within the consolidation process may fully prevent the development of lasting (e.g., neuropathic) pain. For example, when neuropathic pain is induced in rats by placing a cuff around the sciatic nerve, mechanical hyperalgesia develops within 24 h. Removal of the cuff 24 h but not 4 days after implantation allows full recovery of mechanical thresholds within 18 days [21]. Likewise, a single intrathecal injection of GABA apparently reverses neuropathic pain permanently when given 1 week after a chronic constriction injury of the sciatic nerve in rats, but not when given more than 2–3 weeks after the injury [22].

#### Maintenance phase

Most if not all types of lasting pain are associated with distinct forms of functional and morphological remodelling of the nociceptive nervous system. As with all forms of neuronal plasticity, remodelling of the nociceptive system needs to be actively maintained. Specific interference with the mechanisms of maintenance should permanently or temporarily improve or eliminate chronic pain without affecting the properties and functions of acute pain.

## Memory traces of fear

Excessive fear is a characteristic of anxiety disorders. Moreover, it can be argued that there is a link between a specific aversive experience and the resultant anxiety for several disorders. This resembles the chronic pain that can develop after an episode of acute pain. For example, posttraumatic stress disorder (PTSD) emerges following a traumatic episode and specific phobias may be driven, at least in part, by prior aversive learning, both individual and socially mediated. Even compulsive behaviours in obsessive compulsive disorder (OCD) [23] and food avoidance behaviour in anorexia nervosa may arise from abnormalities in fear learning and memory [24]. Therefore, erasing fear memories, or damping down their expression, is thought to be of clinical benefit in these conditions.

### Acquisition, consolidation, and long-term persistence of fear memories

**Figure 2:**
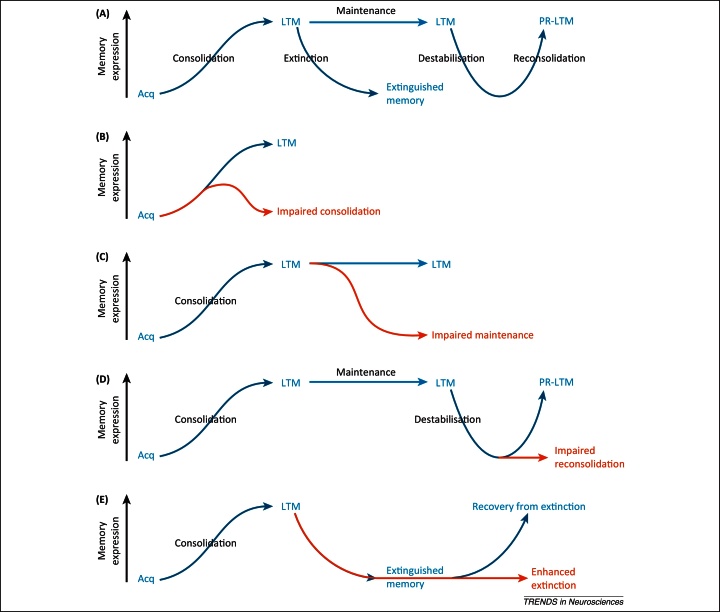
Phases of fear memory and their modulation to reduce fear expression. Blue arrows/text represent normal memory processes; red arrows/text represent interventions to reduce fear expression. (**A**) Phases of fear memory. Consolidation of a memory after its acquisition (Acq) stabilises the long-term memory (LTM) and thereby increases memory expression. Mechanisms of memory maintenance enable the consolidated memory to persist. Reactivation of a LTM can lead to its destabilisation, necessitating a process of reconsolidation to restabilise the memory again into a persistent long-term form (post-reactivation LTM, PR-LTM). Exposure to fear stimuli in the absence of the aversive outcome results in extinction that suppresses memory expression. (**B**) Impairment of consolidation to erase fear memories. Interference with the cellular mechanisms that are required to consolidate a newly acquired memory can prevent the formation of LTM; the memory trace instead decays, leading to reduced memory expression. (**C**) Impairment of memory maintenance to erase fear memories. Interference with the cellular mechanisms of memory maintenance leads to rapid erasure of the memory and hence decay of memory expression. (**D**) Impairment of reconsolidation to erase fear memories. Interference with the cellular mechanisms of memory reconsolidation prevents a destabilised memory from being successfully restabilised; the destabilised memory instead decays, leading to reduced memory expression. (**E**) Enhancement of extinction as a compensatory mechanism to reduce fear. An extinguished memory normally recovers easily. However, pharmacological enhancement of extinction results in a persistent reduction in fear memory expression that appears not to recover.

In the context of clinically relevant fear and anxiety, the underlying memory is Pavlovian in nature, associating previously neutral stimuli with an aversive outcome such as trauma. Subsequent exposure to those now-conditioned stimuli comes to evoke retrieval of the memory, which results in the state of fear and anxiety. As for pain, there are several distinct phases of processing of fear memories (Figure 2A).

The acquisition of fear memories occurs at the time of the aversive experience, probably engaging associative LTP-like processes that connect the previously neutral stimulus with the aversive outcome. Consolidation of this new memory at the cellular level into a long-lasting form takes place over a relatively short timescale (minutes to hours), a restricted time window in which to affect fear memory processing and stabilisation. Although there are undoubtedly more long-lasting memory-processing mechanisms, such as systems-level consolidation as originally hypothesised for hippocampus-related context memories, it is only recently that the time-dependent recruitment of secondary sensory cortices in the very long-term storage of Pavlovian-cued fear memories has been identified [25]. Therefore, this review focuses on those mnemonic processes that are of established relevance to amygdala-dependent fear memories (Box 3).

Once a memory is established, has been consolidated, and has entered the maintenance phase, its expression remains subject to modulation. For Pavlovian fear memories, subsequent learning that the conditioned stimuli are no longer associated with the aversive outcome (i.e., extinction training or exposure therapy) diminishes memory expression. However, the reduction in fear is not necessarily permanent and the fear can be expressed again relatively easily [26]. A second way in which long-term memories are modulated involves the process of memory reconsolidation. Memory retrieval can destabilise the memory, requiring that it be re-established through reconsolidation to persist into the future [27]. What this reconsolidation process appears to allow is an updating of the memory with new information presented at the time of the destabilising retrieval [28].

As for pain, any disruption of the consolidation or maintenance of fear memories, or modulation of their extinction or reconsolidation for therapeutic benefit, must be done selectively. It would not be beneficial to impair the subsequent capacity to form adaptive fear memories or to cause widespread memory loss that would impair other normal behaviours.

## Erasing memory traces of pain and fear

### Erasure of pain memory traces

#### Opioid-induced depotentiation (OID)

Conditioning low-frequency stimulation (LFS) of primary afferents at C-fibre intensity induces LTP at C-fibre synapses and modifies the phosphorylation state of AMPA receptors as described in Box 3. Thus, the specific reversal of these postsynaptic memory traces of pain may comprise normalising the phosphorylation state of AMPA receptors in the spinal dorsal horn and, indeed, it has been recently discovered that this can be achieved [29]. When applied briefly (1 h) at a very high dose, the ultrashort-acting μ-opioid receptor agonist remifentanil normalises the phosphorylation state of AMPA receptors after LFS, dephosphorylating GluR1 at Ser831 and phosphorylating GluR2 at Ser880 [29]. Importantly, when remifentanil is given at the same dose, it not only reverses LFS-induced LTP, capsaicin-induced mechanical hyperalgesia is also partially reversed in behaving animals after washout of the drug [29].

The consolidation phase of LTP begins as early as 3 h after induction and involves *de novo* synthesis of synaptic proteins. When given 4 h after conditioning LFS (i.e., within the consolidation phase of LTP) a single high opioid dose partially reverses LTP. The mechanisms involved in OID during the induction phase of LTP differ from OID in the consolidation phase because the former but not the latter is blocked by calyculin A, an inhibitor of PP1 [29]. Unsurprisingly, pretreatment with the *de novo* protein synthesis inhibitor cycloheximide or anisomycin reduces late-phase LTP (>3 h) without affecting early-phase LTP at spinal C-fibre synapses after conditioning stimulation [30]. Signalling pathways that are required for the active reversal of LTP in nociceptive pathways are shown in Figure 1B. Interestingly, both inhibition of protein synthesis and the classical μ-opioid receptor agonist morphine interfere with the development of PTSD (see below).

#### Counterstimulation

LTP at synapses of sciatic nerve C-fibres can also be depotentiated by conditioning sciatic nerve stimulation at Aδ-fibre intensity. At this stimulation intensity, few or none of the high-threshold C-fibres are recruited, suggesting that depotentiation is likely to involve a heterosynaptic mechanism. The effective Aδ-fibre stimulation protocol resembles some forms of counter-irritation, such as high-intensity transcutaneous electrical nerve stimulation or electroacupuncture [14].

#### D-cycloserine (DCS)

The partial NMDA receptor agonist DCS is used to facilitate inhibition of fear memories (see below) and reduces neuropathic pain-related behaviour when injected directly into limbic areas (medial prefrontal cortex or amygdala) of rats. The effect outlasted the treatment for weeks [31]. Interestingly, in a patient with refractory orofacial pain, transcranial direct-current stimulation over the hand motor area led to a 60% reduction of pain for at least 6 weeks when therapeutic stimulation was combined with DCS [32]. Finally, DCS also reduces pain-related behaviour in the second phase of the formalin test, which is believed to involve LTP at C-fibre synapses [33].

#### Protein kinase M zeta (PKM ζ)

PKM ζ is a persistently active isoform of atypical protein kinase C that potentiates postsynaptic AMPA receptor-mediated currents by enhancing the number of GluR2-containing, active AMPA receptor channels [34]. PKM ζ is sufficient and required to maintain LTP at some synapses in the CNS. Correspondingly, PKM ζ is also necessary for maintaining distinct types of declarative and procedural memory, including fear memory [35–38]. The role of PKM ζ in memory traces of pain depends on the nature of the pain and the location of the synapses involved. Blocking PKM ζ by the inhibitor zeta-pseudosubstrate inhibitory peptide (ZIP) in the spinal cord prevents formalin-induced nociceptive behaviour and reduces mechanical and thermal hyperalgesia after intraplantar administration of Complete Freund's Adjuvant [39] or capsaicin [40]. Spinal application of ZIP also blocks interleukin (IL)-6-induced priming of mechanical hyperalgesia by intraplantar prostaglandin E_2_ injections [41], but does not appear to affect neuropathic pain [42,43]. By contrast, PKM ζ inhibition in the anterior cingulate cortex of mice reverses mechanical hyperalgesia after ligation of the common peroneal nerve [42] and, importantly, also reverses averseness to pain after spinal nerve ligation, as assessed by a conditional place-preference test in rats [43]. Interestingly, blockade of spinal PKM ζ had no effect on spinal nerve ligation-induced aversiveness. Likewise, spinal nerve ligation did not induce PKM ζ-dependent forms of LTP at Aδ- or C-fibre synapses in the spinal dorsal horn [42]. Taken together, the studies published to date suggest that spinal PKM ζ is essential for maintaining nociceptor pain, whereas PKM ζ in the anterior cingulate cortex is required for the expression of evoked and spontaneous neuropathic pain.

#### Glial cells

Growing evidence demonstrates that glial cells are activated in various animal models of chronic pain [44,45]. Activated glial cells then release numerous proinflammatory gliotransmitters that contribute to the amplification of nociception, both by enhancing excitation and by reducing normal inhibition. On activation, the glia switch phenotype from housekeeping cells to pronociceptive helper cells [46], a switch that constitutes another memory trace of pain and can apparently be reversed or compensated by activation of spinal adenosine A2 receptors that are expressed on spinal glial cells. A single intrathecal injection of an adenosine A2 receptor agonist 10–14 days after a chronic constriction nerve injury reverses mechanical and thermal hyperalgesia for at least 4 weeks without affecting normal nociception [47]. When neuropathy is induced by inflammation of the sciatic nerve, minocycline can attenuate allodynia 1 day but not 1 week later [48], suggesting that, for different neuropathic aetiologies, glial cell activation has distinct roles in the maintenance and/or consolidation phases of neuropathic pain.

#### Impaired inhibition

Inhibition serves five important functions in the nociceptive system, as outlined in Table 1 and reviewed in [17,46]. Potassium chloride cotransporter 2 (KCC2) is required for a normal anion gradient across neuronal cell membranes by keeping Cl^−^ concentrations low, a prerequisite for Cl^−^ influx into, and thus the inhibition of, neurons on the opening of GABA_A_ or glycine receptors. The lasting downregulation of KCC2 is another memory trace of pain. Neuropathy leads to the activation of spinal glial cells and to the release of brain-derived neurotrophic factor (BDNF), which results in the downregulation of KCC2 [49,50]. This impairs normal inhibition by GABA_A_ or glycine receptors and thus impedes some or all of the five essential functions of inhibition in the nociceptive system. Blockade of microglial function by minocycline or BDFN function by TrkB/Fc restores KCC2 levels and reverses neuropathic pain [51].

### Erasure of fear memory traces

Given that LTP is also thought to be a fundamental mechanism of memory encoding in the amygdala, which is a critical locus of fear memories [52,53], reversing the plasticity underlying LTP would result in the erasure of fear memory traces. This reversal might be achieved in several ways, although it is functionally difficult to determine conclusively that a memory has been erased. Rather, from a clinical perspective, an outcome in which the expression of the fear memory is diminished in a meaningful and long-lasting manner is desirable.

Although the treatment of anxiety disorders would canonically be started once a patient presents with diagnosed symptoms, the first stage at which a fear memory might be erased is at the time of acquisition and consolidation (Figure 2B). Unlike the prevention of pain (e.g., attempts by pre-emptive analgesia), the prevention of fear memory acquisition is an unlikely approach, because there would be no obvious way prophylactically to discriminate between adaptive and problematic memories. However, given that the formation of long-term fear memories requires a post-acquisition consolidation process, impairment of consolidation is a viable approach, particularly because the newly formed memory remains vulnerable to disruption for up to 6 h following its acquisition. Treatment of victims of trauma with the beta-blocker propranolol or morphine in the immediate aftermath of the traumatic incident appears to reduce the likelihood of them developing PTSD [54–57]. However, other studies have failed to replicate the propranolol findings in different clinical settings [58,59] and although targeting memory consolidation may be a viable therapeutic strategy, in this review we concentrate on retrospective treatment approaches.

The first such approach, and perhaps the closest to true erasure, involves the disruption of memory maintenance (Figure 2C). This differs conceptually from the prevention of memory consolidation in the first place. Although there are many mechanisms that have been identified as being important for memory consolidation, only one drug thus far appears to reverse LTP and erase memories. Much as in the nociceptive system, the persistence of hippocampal synaptic potentiation in rodents is critically dependent on the activity of the atypical protein kinase PKM ζ and inhibition of PKM ζ by the inhibitory peptide ZIP seemingly both reverses LTP [60] and erases hippocampal memories [61]. ZIP blocks the maintenance of late-phase LTP in the amygdala [42] and at the behavioural level infusions of ZIP directly into the amygdala disrupt fear memories in rats [37,62]. Whether this disruption reflects erasure of the fear memory remains unclear, because the expression of fear recovered despite ZIP infusion in a fear-potentiated startle setting [38]. Moreover, it is now unclear whether ZIP exerts its memory-disrupting effects only through inhibition of PKM ζ, because ZIP has similar amnestic effects in mice that have been genetically modified not to express PKM ζ [63,64]. Finally, from a translational perspective, inhibition of PKM ζ may not be a viable treatment strategy for fear memories. Studies of cortical memories reveal that ZIP appears to disrupt all plasticity within the target region [65] and the amygdala is critical not only for maladaptive fear, but also for normal fear responses and reward-related Pavlovian memories [66]. Therefore, although PKM ζ inhibition may successfully erase problematic fear memories, it is also likely to have detrimental effects on normal adaptive behaviour.

Given the potential for detrimental effects arising from attempts to erase fear memory traces, the selectivity of memory disruption becomes a critical consideration. This seems to be a potential advantage for an approach based on disrupting memory reconsolidation (Figure 2D). Retrieval of a fear memory can initiate a process of memory reconsolidation that is necessary for re-establishment and subsequent retrieval [27]. For Pavlovian fear memories in rats, reconsolidation was first disrupted by infusions of the protein synthesis inhibitor anisomycin into the amygdala [67]. Importantly, it is only the memory trace successfully reactivated by memory retrieval that is disrupted by anisomycin [68]. Moreover, the disruption of fear memory expression was associated with a reduction in synaptic potentiation [68], indicating that the memory trace was erased. Although inhibition of local protein synthesis is not likely to be of translational utility, its disruptive effect can be replicated by systemic administration of drugs such as the NMDA receptor antagonist MK-801 [69] and the beta-blocker propranolol [70], the latter also having promising effects in healthy human studies [71] and an open-label trial in PTSD patients [72]. Perhaps even more exciting is the potential for a purely behavioural retrieval–extinction strategy based on reconsolidation processes that diminishes the reactivated memory with stimulus exposure within the so-called ‘reconsolidation window’ of plasticity. Initially demonstrated in rats [73], studies in human fear conditioning have demonstrated the selectivity of the approach [74]. However, questions remain over its translational efficacy. When fear-relevant stimuli, such as pictures of spiders, were used as conditioned stimuli to heighten levels of fear, the expression of fear was only transiently diminished by retrieval–extinction [75], contrasting with the persistent reduction observed when propranolol was administered to disrupt fear memory reconsolidation [76]. Secondly, the retrieval–extinction procedure appears to disrupt relearning of the fear memory [73], potentially leaving a treated individual less able to form adaptive fear memories.

## Compensating for – rather than reversing – pathological changes

Much is known about the distinct pathological changes that contribute to different forms of lasting pain. As outlined above, our knowledge about the true reversal of these changes is only beginning to evolve. Attempts have been made to compensate for pain-related changes within the CNS and successful interference at different levels of the neuraxis is illustrated by the following examples. Descending facilitation via activation of monoaminergic pathways contributes to some forms of opioid-induced hyperalgesia and to neuropathic pain. Reduction of noradrenergic input to the dorsal reticular nucleus, which is part of the pronociceptive circuitry, by injections of a viral vector that carried the tyrosine hydroxylase transgene in antisense orientation, 2 weeks after spinal nerve injury, induces long-lasting attenuation of mechanical hyperalgesia [77].

Neuropathic pain leads to the release of proinflammatory cytokines from glial cells in the spinal dorsal horn, partly by continuous activation of toll-like receptor 4, a pattern-recognition receptor expressed by glial cells. Blocking the ongoing activation of this receptor by systemic injection of (+)-naloxone 2–4 months after spinal nerve ligation or chronic constriction injury of the sciatic nerve temporarily reverses mechanical hyperalgesia [78].

Convincing evidence suggests that impaired GABAergic inhibition in the spinal dorsal horn contributes to various forms of inflammatory and neuropathic pain [17,79]. Continuous pharmacological activation of spinal GABA_A_ receptors containing the α_2_ and/or α_3_ subunit may compensate for insufficient endogenous GABAergic tone in spinal nociceptive circuits, which dampens inflammatory and neuropathic hyperalgesia without affecting normal nociceptive responses [80].

Enhancing inhibitory processes in the treatment of pain bears striking conceptual similarity to the potential for enhancing fear memory extinction to reduce anxiety. Exposure to fearful stimuli in the absence of an aversive outcome results in memory extinction that suppresses fear expression. However, exposure therapy has limited benefits due to the propensity for recovery of fear responses. Therefore, pharmacological enhancement of the extinction process may be of further benefit (Figure 2E). There are several agents that potentiate fear memory extinction in experimental animals, including methylphenidate [81] and fibroblast growth factor-2 [82]. However, the most-studied drug is the NMDA receptor partial agonist DCS.

When DCS is administered either systemically or directly into the amygdala, it potentiates extinction quantitatively to produce a greater reduction in fear memory expression [83,84]. Importantly, the enhancement of extinction achieved by DCS is also qualitative, with there being no evidence of memory recovery following normally effective reminder procedures [84,85]. This reduction in the recovery of fear suggests that DCS might achieve a meaningful and long-lasting reduction in fear in anxiety disorders; indeed, initial studies do show beneficial effects in various anxiety disorders [86–91]. However, DCS did not benefit a population of participants who were fearful of spiders at a non-clinical level [92] nor did it produce an obvious reduction in fear in the context of PTSD [93,94]. Moreover, preclinical studies in rodents revealed that DCS has no effect when a fear memory is being re-extinguished [95]. Finally, and potentially more problematically, it is known for fear memories that there is competition between reconsolidation and extinction. Given that reconsolidation and extinction share many cellular mechanisms; attempts to disrupt reconsolidation might instead impair extinction, maintaining the expression of fear memories. Conversely, administration of DCS with the intention of potentiating extinction might enhance reconsolidation to strengthen the fear memory, as has been observed in rodent fear models [69]. Therefore, interventions based on compensation are not likely to complement those aimed at true memory erasure.

## Concluding remarks

The mechanisms that lead to and maintain chronic pain are fundamentally different from those relevant to acute pain. Most currently employed pharmacotherapies are, however, directly derived from animal models of acute nociceptor and inflammatory pain. This includes the continuous application of a moderate opioid dose and non-steroidal anti-inflammatory drugs. Thus, it is unsurprising that these therapies work well for acute pain but are largely ineffective in curing chronic pain. The true reversal of the pathological changes that contribute to chronic pain require in-depth understanding of the distinct mechanisms that operate during the induction, consolidation, and maintenance phases of the various types of lasting pain. Similarly, the potential reversal of aberrant fear memories draws on understanding of the mechanisms of memory maintenance and reconsolidation. Interference with the induction phase of aversive memory trace formation is more relevant to the management of pain than fear. However, such pre-emptive therapy must not be restricted to analgesics, because increased acute nociception is not the sole trigger for the development of chronic pain. At present, it appears most promising to interfere during the consolidation phase of chronic pain to prevent long-term plasticity in the nociceptive system and chronic pain. By contrast, a focus on fear memory reconsolidation is the most promising approach to interfere selectively with long-term fear plasticity. Finally, compensation for pathological changes is an option applicable to both fear and pain and in the latter appears to be superior to the application of conventional analgesics for chronic pain. However, there is clearly a great demand for true reversal of the long-lasting plasticity that underlies chronic pain and anxiety; that is, to erase memory traces of pain and fear. Understanding of how to achieve this goal is only just emerging.

## Figures and Tables

**Table 1:** The five roles of inhibition in pain control

Role of inhibition	Mechanism of action	Desired effect	Pain type on failure
Muting	Inhibition of nociceptive dorsal horn neurons and interneurons driving those	Silencing principal pain neurons in the absence of noxious stimuli	Spontaneous pain
Attenuation	Pre- and postsynaptic inhibition of nociceptive spinal dorsal horn neurons	Proper response level of principal pain neurons to noxious stimulation	Hyperalgesia
Limiting	Inhibition of excitatory interneurons that cross somatotopic borders	Limiting spread of excitation to somatotopically appropriate areas	Radiating pain, referred pain, mirror-image pain
Separating	Inhibition of excitatory interneurons linking Aβ-fibre input to nociception-specific neurons	Inhibition of excitatory crosstalk between sensory modalities	Allodynia
Preventing	Reduced Ca^2+^ influx into nociceptive spinal dorsal horn neurons	Blocking Ca^2+^-dependent signalling pathways engraining memory traces of pain	Chronic pain

The table summarises the five principle functions of inhibition in the nociceptive system (modified) [46].

## Box 1 Staged formation of memory traces

Memory is the retention of information that modifies future behavioural and/or neuronal responses. A fundamental feature of information processing in the CNS is the capacity to store information by long-term changes of synaptic strength, and synaptic LTP is a much-studied cellular model of learning and memory formation [17,36,53,96–98]. The formation of memory is a staged process that involves the acquisition (or induction) phase, the consolidation phase, and the maintenance phase. The initial experience that is to be encoded induces an acquisition process that by itself is insufficient to form a long-lasting trace. Rather, the memory must be consolidated following acquisition to stabilise it into a long-term form. Many cellular mechanisms have been shown to be involved in this process, including the necessity for protein synthesis [99]. The vulnerability of new memories to protein synthesis inhibition lasts for only a short period following acquisition [100]. However, the long-term persistence of the memory continues to depend on cellular mechanisms of memory maintenance [36]. Much progress has been made in elucidating the distinct mechanisms and signalling pathways that contribute to LTP induction, consolidation, and maintenance; readers are referred to several excellent reviews [17,36,53,96–98]. Considerably less is known about the equally important reversal of synaptic plasticity, such as the depotentiation of LTP. Without continuous and targetted depotentiation, LTP would eventually lead to a state where essentially all synapses in the CNS would be at a saturated level of potentiation, thereby losing all capacity to store novel information. LTP and depotentiation are thus inseparable mechanisms that regulate the formation and storage of memory and the erasure of older or less relevant memory traces. Several pathological conditions may involve a disturbance in the physiological balance between the formation and erasure of memory traces, including dementia, schizophrenia, stress and anxiety disorders, and chronic pain.

## Box 2 Some useful definitions

Principal pain neurons are neurons that lead to the sensation of pain when activated. In the peripheral nervous system nociceptive Aδ- and C-fibres appear to function as principle pain neurons. In the CNS, principle pain neurons remain to be identified with certainty, but neurons in spinal cord lamina I that express the neurokinin 1 receptor and project to the brain are good candidates for pain under pathological conditions [46,101].

The neuronal mechanisms contributing to lasting pain are often collectively called memory traces of pain. This must not be confused with the explicit memory of a previous pain experience; that is, the recollection of its location, nature, intensity, or duration as surveyed during the taking of a medical history. Similarly, there is a distinction between unconscious Pavlovian fear memories that underlie both adaptive and maladaptive behaviours and the conscious declarative recollection of the aversive or traumatic episode. For both pain and fear, it is the unconscious memory that is the target of therapeutic intervention.

Analgesia is an umbrella term for all forms of pain relief. Antihyperalgesia refers to forms of analgesia that are effective in the presence of hyperalgesia, but either not effective or less effective for physiological pain. Antihyperalgesia requires interference with (neuro-)plastic changes underlying the generation or amplification of pain. The erasure of a memory trace of pain implies that at least one of the neuronal causes of lasting pain is eliminated. This does not necessarily reverse pain permanently, because new memory traces of pain may be formed due to an ongoing disease such as a neuropathy or chronic inflammation.

Erasure of a memory trace of fear would result from a long-lasting disruption or reversal of the neuronal mechanisms supporting the fear memory. Although the amygdala is the most obvious target for fear memory disruption, synaptic plasticity in several other neural loci also play important roles in fear memories [63].

## Box 3 LTP in neuronal networks for pain and fear

Pain perception and fear involve complex, partially overlapping neuronal networks that can in principle be modulated at all synaptic relay stations involved. Long-term plasticity has been characterised most extensively at C-fibre synapses in the superficial spinal dorsal horn [17], at cortical synapses including the anterior cingulate cortex [102], and in the amygdala [68]. LTP at some of these relays can thus affect and mutually interfere with both pain and fear. LTP at multiple sites in serially connected pathways (e.g., in spinal cord and in the anterior cingulate cortex) can function as a ‘cascade amplifier’ boosting signal amplification at each relay station.

*LTP in nociceptive pathways*

Glutamate is the neurotransmitter at the first synaptic relay in nociceptive pathways between C-fibre afferents and neurons in the superficial spinal dorsal horn. Excitation of postsynaptic neurons is largely due to activation of ionotropic AMPA glutamate receptors, but NMDA and kainate receptors also play a role. Synaptic LTP at glutamatergic synapses of C-fibre afferents is an intensively studied neuronal mechanism leading to long-lasting enhanced excitability in nociceptive pathways [17]. LTP can be induced by noxious stimulation, acute nerve injury, electrical nerve stimulation at C-fibre intensity [103–106], abrupt withdrawal from opioids [20], and spinal application of various substances [107–109]. Induction of LTP requires postsynaptic Ca^2+^-dependent signalling pathways, including the activation of protein kinases A and C, Ca^2+^/calmodulin-dependent protein kinase II (CaMKII), and protein phosphatase 1 (PP1) [110].

AMPA receptors can be phosphorylated by these kinases at various sites and can be dephosphorylated by PP1. Protein kinase C- and CaMKII-dependent phosphorylation of the GluR1 subunit at Ser831 enhances single-channel conductance and its time course closely parallels post-injury pain amplification [111]. By contrast, dephosphorylation of the GluR2 subunit at Ser880 by PP1 leads to reduced endocytosis of AMPA receptors, enhanced receptor density in the postsynaptic membrane, and, consequently, the amplification of glutamatergic currents. A likely memory trace of pain thus comprises the altered phosphorylation status of AMPA receptors leading to enhanced AMPA receptor-mediated currents. Changes in the phosphorylation state of AMPA receptors may underlie postsynaptic expression of LTP at C-fibre synapses, but any additional contribution of presynaptic mechanisms to the expression of LTP at C-fibre synapses must not be ignored [112].

*LTP in fear memory pathways*

The synaptic mechanisms of LTP in fear-relevant neural loci are much the same as those described for nociceptive pathways. LTP has been described for the lateral amygdala (LA) [53] in the synaptic convergence between the sensory afferents carrying stimulus information and the somatosensory afferents carrying the aversive nociceptive information [113]. The former can include both thalamic and cortical sensory pathways [114,115]. However, whereas synaptic plasticity in the LA is critical for fear memory formation, it is not the only locus of LTP engaged by fear learning. Plasticity in the auditory thalamus has sometimes been shown to be necessary for fear conditioning to a tone stimulus, by targeting important cellular mechanisms of LTP such as mitogen-activated protein kinase (MAPK) activation [116,117]. Moreover, protein synthesis-dependent synaptic plasticity in the dorsal and ventral regions of the hippocampus is important for fear conditioning to a contextual stimulus [118,119], suggesting the functional involvement of LTP in those loci. Finally, additional areas are important for fear memory expression. For example, the prelimbic cortex is necessary for the normal expression of fear responses and inhibiting prelimbic activity suppresses fear expression [120]. Similarly, inactivation of the nucleus accumbens impairs both the acquisition and the expression of conditioned fear [121]. However, it remains unclear whether synaptic plasticity is necessary in the prelimbic cortex or nucleus accumbens for learned fear.
